# Associations between depression and cancer risk among patients with diabetes mellitus: A population‐based cohort study

**DOI:** 10.1002/cam4.6539

**Published:** 2023-09-14

**Authors:** Wang Shi‐Heng, Le‐Yin Hsu, Mei‐Chen Lin, Chi‐Shin Wu

**Affiliations:** ^1^ National Center for Geriatrics and Welfare Research National Health Research Institutes Miaoli Taiwan; ^2^ Department of Public Health, College of Public Health China Medical University Taichung Taiwan; ^3^ Institute of Epidemiology and Preventive Medicine, College of Public Health National Taiwan University Taipei Taiwan; ^4^ Graduate Program of Data Science National Taiwan University and Academia Sinica Taipei Taiwan; ^5^ Department of Psychiatry National Taiwan University Hospital, Yunlin Branch Douliu Taiwan

**Keywords:** antidepressant, cancer, cohort, depression, diabetes

## Abstract

**Background:**

The co‐occurrence of depression and diabetes mellitus has been linked to an increased risk of developing cancer. This study aimed to investigate whether depression further amplifies the risk of cancer among individuals with diabetes.

**Methods:**

This population‐based matched cohort study utilized Taiwan's National Health Insurance claims database. A total of 85,489 newly diagnosed diabetic patients with depressive disorders were selected, along with 427,445 comparison subjects. The matching process involved age, sex, and the calendar year of diabetes onset. The average follow‐up duration for the two cohorts was 6.4 and 6.5 years, respectively. The primary outcome of interest was the occurrence of overall cancer or cancer at specific anatomical sites.

**Results:**

The adjusted hazard ratios for overall cancer incidence were 1.08 (95% CI, 1.05–1.11). For site‐specific cancers, depression exhibited significant associations with oropharyngeal, esophageal, liver, gynecological, prostate, kidney, and hematologic malignancies among patients with diabetes. Notably, a severity‐response relationship was observed, indicating that patients with recurrent episodes of major depressive disorders exhibited a higher incidence of cancer compared to those diagnosed with dysthymia or depressive disorder not otherwise specified. Furthermore, the strength of the association between depression and cancer risk was more pronounced among younger patients with diabetes as opposed to older adults. However, no significant relationship was observed between adherence to antidepressant treatment and cancer risk.

**Conclusions:**

The findings of this study indicate a significant association between depression and an elevated risk of cancer among individuals diagnosed with diabetes. Future investigations should replicate our findings, explore the effects of pharmacological and non‐pharmacological treatments on cancer risk, and identify the underlying mechanisms.

## 1 BACKGROUND

Diabetes mellitus is an increasing and highly prevalent disorder, affecting an estimated 463 million people globally in 2019.^1^ Numerous adverse health outcomes have been associated with diabetes, including mortality, cardiovascular disorders, microvascular complications, and cancer risk.^2^, ^3^

Depression is a widely prevalent mental disorder, with its occurrence varying across different countries. The lifetime prevalence of depression ranges from 19.2% in the United States to 6.6% in Japan.^4^ Notably, depression frequently co‐occurs with diabetes, a prevalent medical condition. The prevalence of depression among patients with diabetes mellitus surpasses that observed in individuals without diabetes.^5^ Moreover, prior research has established bidirectional associations between the incidence of depression and diabetes mellitus.^6^, ^7^

The coexistence of depression and diabetes has been recognized as contributing to adverse outcomes and increased mortality in individuals with diabetes.^8^, ^9^ Previous research has indicated a potential association between depression and an elevated risk of cancer development.^10^, ^11^ Additionally, diabetes has been identified as an independent risk factor for cancer incidence.^12^ Whether the coexistence of depression and diabetes further increases cancer risk has not been fully explored. One study reported an association between major depression and increased all‐cause mortality but not cancer‐specific mortality among patients with diabetes.^13^ Another study found no significant association between depressive symptoms and cancer incidence, but the limited sample size may have influenced the null findings.^14^ Prior demonstrations of severe depressive symptoms have been linked to suboptimal diabetes control.^15^ Psychological distress has exhibited an association with increased mortality, especially among individuals with diabetes.^16^ However, the potential influence of symptom severity on the association between the coexistence of depression and diabetes and its relationship with cancer risk remains inadequately elucidated. Furthermore, antidepressants as the standard treatment for depressive disorders may mitigate the adverse outcomes of depression‐related diabetic complications.^17^ Depression severity has been linked to adherence to both antidepressant and antidiabetic medications.^18^, ^19^ Additionally, research suggests that antidepressants could potentially mitigate cancer risk.^20^, ^21^ Furthermore, a solid adherence to antidepressant treatment has been correlated with decreased mortality among cancer patients within a specific cohort.^22^ However, a systematic review of preclinical in vivo investigations indicated a potential carcinogenic effect of antidepressants.^23^ Nevertheless, recent meta‐analyses based on observational studies have not established significant associations between antidepressant use and specific cancer types.^24^, ^25^ The impact of diabetes on the association between antidepressant adherence and cancer risk remains uncertain.

This matched cohort study aims to explore: (1) the associations between depressive disorders and overall as well as site‐specific cancer risks among individuals diagnosed with diabetes mellitus; (2) the potential modifying effects of sex and age on the link between depressive disorders and cancer risks; (3) the associations between cancer risk and depressive disorder severity; (4) the relationship between cancer risk and antidepressant medication adherence.

## 2 METHODS

Ethical approval for this study was granted by the Institutional Review Board of the China Medical University Hospital (CMUH105‐REC1‐079 CR‐4). Informed consent was waived, as the personal information contained in the National Health Insurance claims database was encrypted.

### 2.1 Data source

This population‐based matched cohort study utilized the claims database from Taiwan's National Health Insurance (NHI) program, which encompasses approximately 99% of the Taiwanese population, totaling 23 million individuals. The claims database provided access to patient's demographic information, clinical diagnoses, and prescription records. The reliability of the diagnostic codes for depressive disorder and diabetes mellitus in the NHI claims database has been extensively validated in previous studies.^26^, ^27^ The NHI claims database has been widely employed in cancer epidemiology research.^28^

### 2.2 Study population

Utilizing the NHI claims database, we included adult patients (aged ≥18 years) who had a clinical diagnosis of diabetes mellitus (International Classification of Diseases, Ninth Revision [ICD‐9] code: 250) with anti‐diabetes treatment (coded as A10 in the Anatomical Therapeutic Chemical classification) between 2001 and 2012, resulting in a cohort of 1,622,639 individuals. The date of the first prescription for antidiabetic medication was considered the onset of diabetes. To ensure the study's internal validity, specific exclusions were applied. These comprised (1) patients with a preexisting cancer diagnosis prior to the commencement of diabetes (*n* = 151,858); (2) individuals with discrepancies in their recorded sex, birthday, or mortality date details (*n* = 12,805); and (3) subjects who experienced mortality (*n* = 47,790) or received a cancer diagnosis within 1 year after the onset of diabetes (*n* = 23,673). This approach ensured a minimum one‐year latent period. Ultimately, the analysis encompassed a cohort of 1,386,513 patients with incident diabetes.

### 2.3 Cohort of patients with incident diabetes and depressive disorders

Patients with depressive disorders, encompassing major depressive disorders with recurrent episodes (ICD‐9 code: 296.3), major depressive disorder with a single episode (ICD‐9 code: 296.2), dysthymic disorder (ICD‐9 code: 300.4), and depressive disorder not otherwise specified (NOS; ICD‐code: 311), were identified prior to the onset of diabetes. A total of 85,489 individuals with comorbid diabetes and depressive disorders were identified. We did not consider individuals who developed depressive disorders after the diabetes diagnosis into the depression group to mitigate immortal time bias. This arises because this subgroup must survive without a cancer diagnosis until the depression is diagnosed. Including these patients as comparisons could result in misclassifications. Hence, 72,503 patients who developed depressive disorders after the onset of diabetes were excluded.

### 2.4 Comparison cohort of patients who had diabetes without depressive disorders

Comparison subjects who did not receive a diagnosis of depressive disorder during the study period were identified. For each patient with concurrent depression and diabetes, five comparison participants were randomly selected, matched by birth year, sex, and the calendar year of diabetes onset. Ultimately, the analysis included 427,445 patients with diabetes and without a diagnosis of depressive disorder.

### 2.5 Main outcome measures

Overall, incident cancer was identified based on at least two ambulatory or one inpatient claim record with diagnostic codes (coded as ICD‐9‐CM: 140–208). Site‐specific cancers were identified using the same criteria, including oropharyngeal cancer (coded as ICD‐9: 140–149), cancer of the nasal cavity and paranasal sinuses (coded as ICD‐9: 160), laryngeal cancer (coded as ICD‐9: 161), esophageal cancer (coded as ICD‐9: 150), stomach cancer (coded as ICD‐9: 151), colorectal cancer (coded as ICD‐9: 153 & 154), liver cancer (coded as ICD‐9: 155), pancreatic cancer (coded as ICD‐9: 157), lung cancer (coded as ICD‐9: 162), breast cancer (coded as ICD‐9: 174 and 175), gynecological cancer (coded as ICD‐9: 179–184), prostate cancer (coded as ICD‐9: 185), bladder cancer (coded as ICD‐9: 188), kidney cancer (coded as ICD‐9: 189), and hematologic malignancy (coded as ICD‐9: 200–203 and 205–208). These site‐specific cancers were categorized based on previous research for cancer associated with depression or diabetes.^28^, ^29^, ^30^ Cancers with a low incidence were not included.

### 2.6 Covariates

The patients' demographic variables, including age, sex, and the calendar year of diabetes onset, were considered. Potential confounding factors associated with both depressive disorder and cancer incidence were evaluated, taking into account comorbid conditions and concomitant medication use in the year prior to the onset of diabetes. Comorbid conditions considered were hypertension (coded as ICD‐9: 401–405), dyslipidemia (coded as ICD‐9: 272), chronic pulmonary disease (coded as ICD‐9: 491–494, 496), alcohol and/or substance use disorder (coded as ICD‐9: 291, 292, 303, 304, 305, excluding 305.1), schizophrenia (coded as ICD‐9: 295), and bipolar disorders (coded as ICD‐9: 296.0–296.1, 296.4–296.8). Concomitant medication use was assessed for angiotensin converting enzyme inhibitor/angiotensin II receptor blockers (coded as ATC: C09), beta‐blockers (coded as ATC: C07), calcium channel blockers (coded as ATC: C08), diuretics (coded as ATC: C03), lipid‐lowering agents (coded as ATC: C10), nonsteroidal anti‐inflammatory drugs (coded as ATC: M10), and antipsychotics (coded as ATC: N05A, excluding N05AN).

Adherence to antidiabetic medication was measured using the medication possession ratio (MPR).^31^


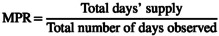




In this study, MPR was calculated by dividing the sum of the days' supply of dispensed medication by the 12‐month observation period following the onset of diabetes. Generally, MPR was defined as good adherence if the MPR ≥0.8.^31^ In order to demonstrate a clear dose–response relationship, we further categorized those MPR <0.8 into poor and partial adherence. Finally, patients were categorized into poor users (0 < MPR < 0.2), partial users (0.2 ≤ MPR < 0.8), and regular users (MPR ≥0.8).

### 2.7 Statistical analysis

The baseline characteristics of patients with incident diabetes, both with and without depressive disorders, were summarized using case numbers and percentages. All study participants were followed up starting from 365 days after the onset of diabetes, defined as the cohort start date, until the occurrence of study outcomes, death, or the end of 2014, whichever came first. Unnatural deaths, considered competing risks in our analysis, were identified through Taiwan's National Death Registry. We conducted a competing risk survival analysis^32^ to estimate the hazard ratio (HR) and 95% confidence interval (CI) between depressive disorders and cancer incidence, including overall and site‐specific cancer. Considering the severity‐response relationship, patients with depressive disorders were further categorized as follows: those with major depressive disorders with recurrent episodes, major depressive disorders with a single episode, dysthymic disorder, and depressive disorder, NOS based on the ICD‐9 code.

The adherence to antidepressant treatment was measured by MPR between the diabetes incidence date and cohort end date. They were classified as poor users (0 < MPR < 0.2), partial users (0.2 ≤ MPR < 0.8), and regular users (MPR ≥0.8).^31^

The modifying effects of age group (18–44, 45–64, and≥ 65 years) and sex were estimated by subgroup analysis. The interaction term between depression and age group or sex was also examined. Statistical analyses were performed using SAS version 9.4 (SAS Institute Inc.). The significance of relationships was evaluated using a 95% confidence interval (CI) or *p*‐value < 0.05.

## 3 RESULTS

### 3.1 Study population characteristics

Most patients with incident diabetes and depressive disorders were female (61.7%), with the age group of 45–64 years representing the highest proportion (48.1%). Compared with the diabetes only cohort, the depression and diabetes cohorts had a higher proportion of hypertension (54.4% vs. 47.6%), dyslipidemia (32.2% vs. 25.8%), comorbid anxiety disorder (33.7% vs. 8.0%), hospitalization in the past year (25.7% vs. 18.6%), and poor use of antidiabetic medication in the year after diabetes onset (33.3% vs. 30.7%; Table S1).

### 3.2 Incident cases of overall cancer and site‐specific cancer

The follow‐up person‐years were 547,747 and 2,783,165, and the mean follow‐up years were 6.4 and 6.5 in the depression and diabetes, and diabetes only cohorts, respectively. There were 8157 incident cancers among patients with coexisting diabetes and depression and 35,867 incident cancers among the comparison subjects. The number of incident cancer of site‐specific cancer and the corresponding follow‐up person‐years are presented in Table S2.

**FIGURE 1:**
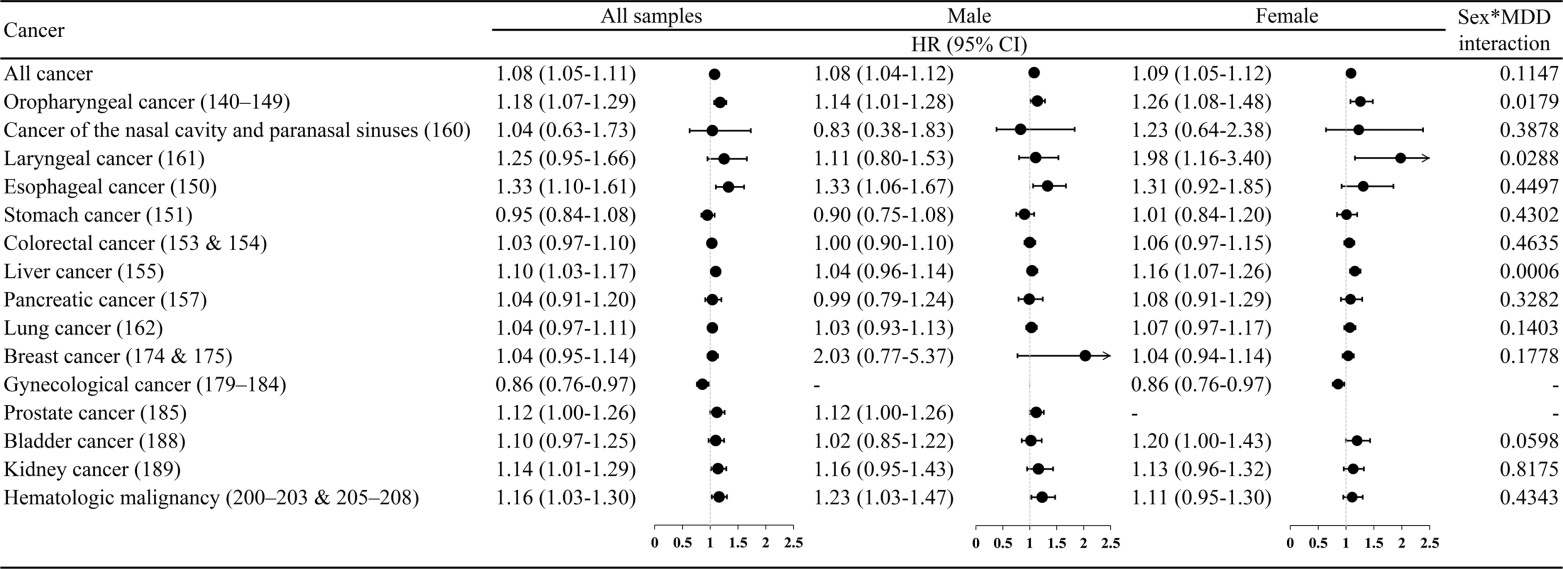
Hazard ratio of all cancers and site‐specific cancer for depression in patients with diabetes.

After adjusting for baseline comorbidity, medication use, health system utilization, and antidiabetic medication MPR, the adjusted HR (95% CI) of incident cancer for patients with depression and diabetes was 1.08 (1.05–1.11) compared to that of the diabetes patients without depression (Figure 1). Considering site‐specific cancer, we found that patients with depression and diabetes had statistically significantly increased risks for oropharyngeal, esophageal, liver, prostate, kidney, and hematologic malignancy.

**FIGURE 2:**
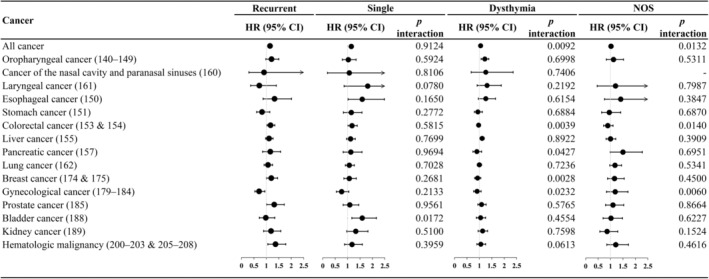
Hazard ratio of all cancers and site‐specific cancer for depression in patients with diabetes, stratified by depression subtype.

Regarding the association between depressive severity and incident cancer (Figure 2, detailed numbers in Table S3), we found that the increased hazard of overall cancer among patients with major depressive disorders with recurrent episodes (adjusted HR = 1.15 [1.08–1.22]) was higher than that among patients with dysthymia (adjusted HR = 1.05 [1.01–1.09], *p*‐value for interaction = 0.0092), and depressive disorders NOS (adjusted HR = 1.02 [0.94–1.11], *p*‐value for interaction = 0.0132). Considering site specificity, such a dose–response association was noted only for colorectal cancer. Of note, we found that gynecological cancer had a reverse association with the severity of depressive disorders.

**FIGURE 3:**
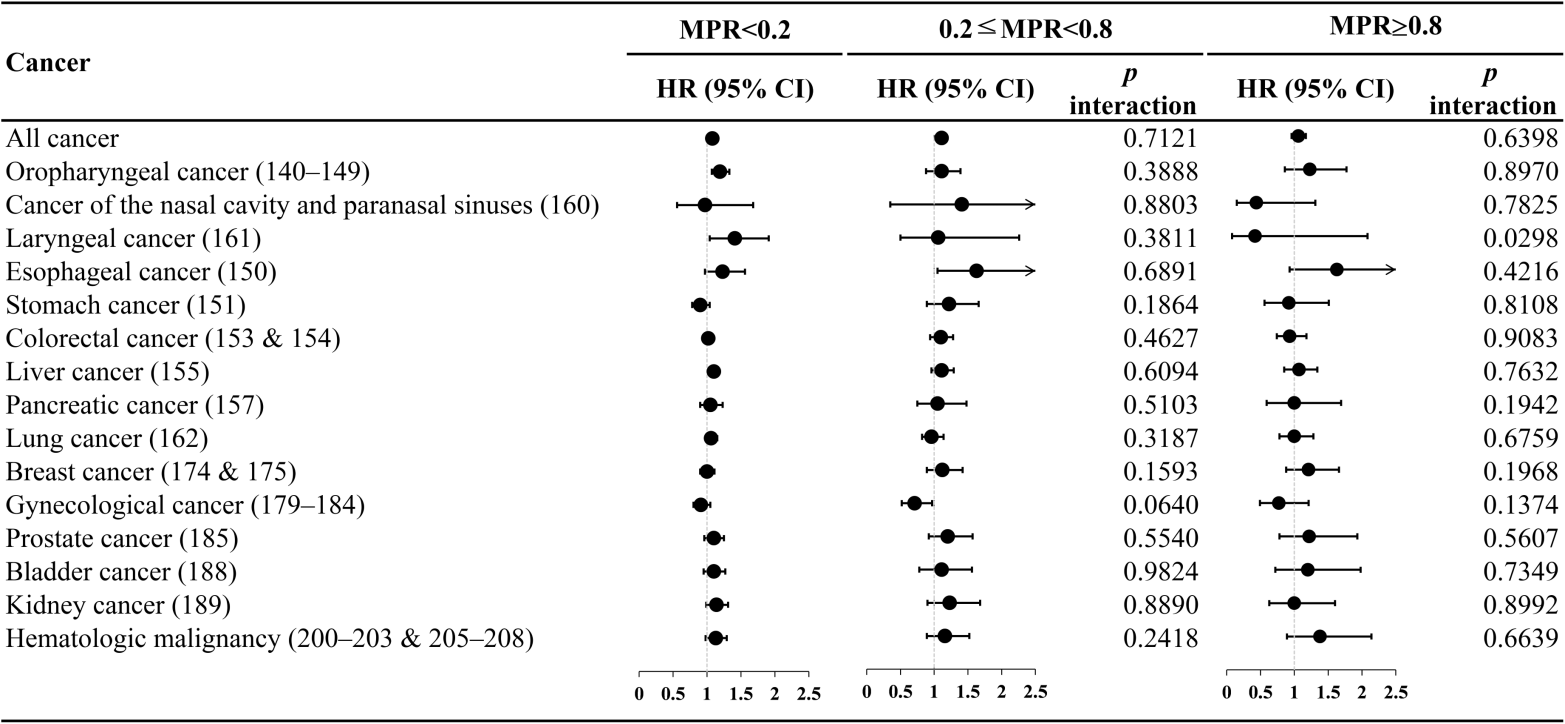
Hazard ratio of all cancers and site‐specific cancer for depression in patients with diabetes stratified by antidepressant MPR.

Regarding adherence to antidepressant treatment (Figure 3, detailed numbers in Table S4), we found no association with cancer incidence, except in patients with poor use of antidepressants who had a higher risk for larynx cancer than those with regular use (*p* = 0.0298).

**FIGURE 4:**
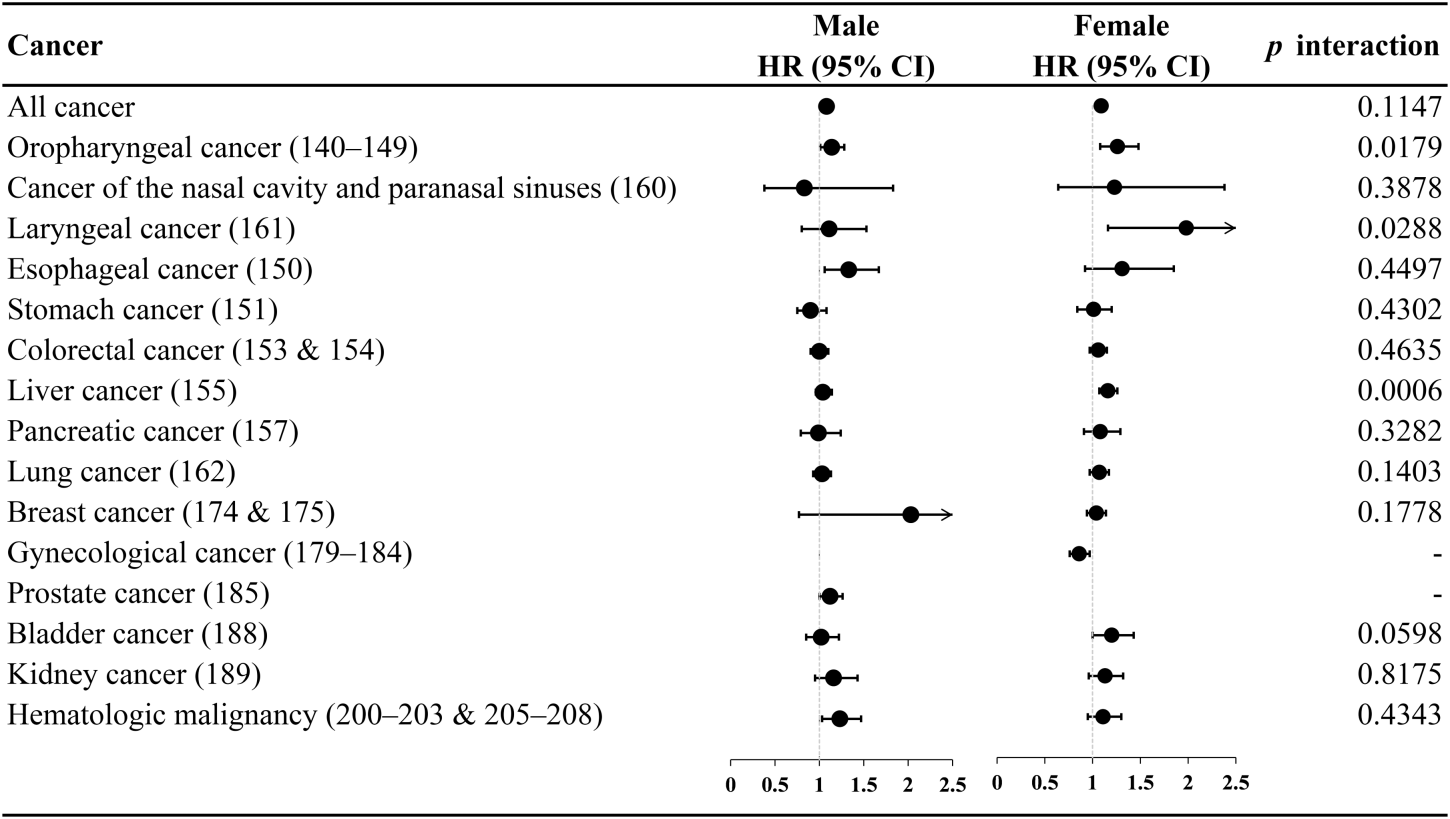
Hazard ratio of all cancers and site‐specific cancer for depression in patients with diabetes, stratified by sex.

**FIGURE 5:**
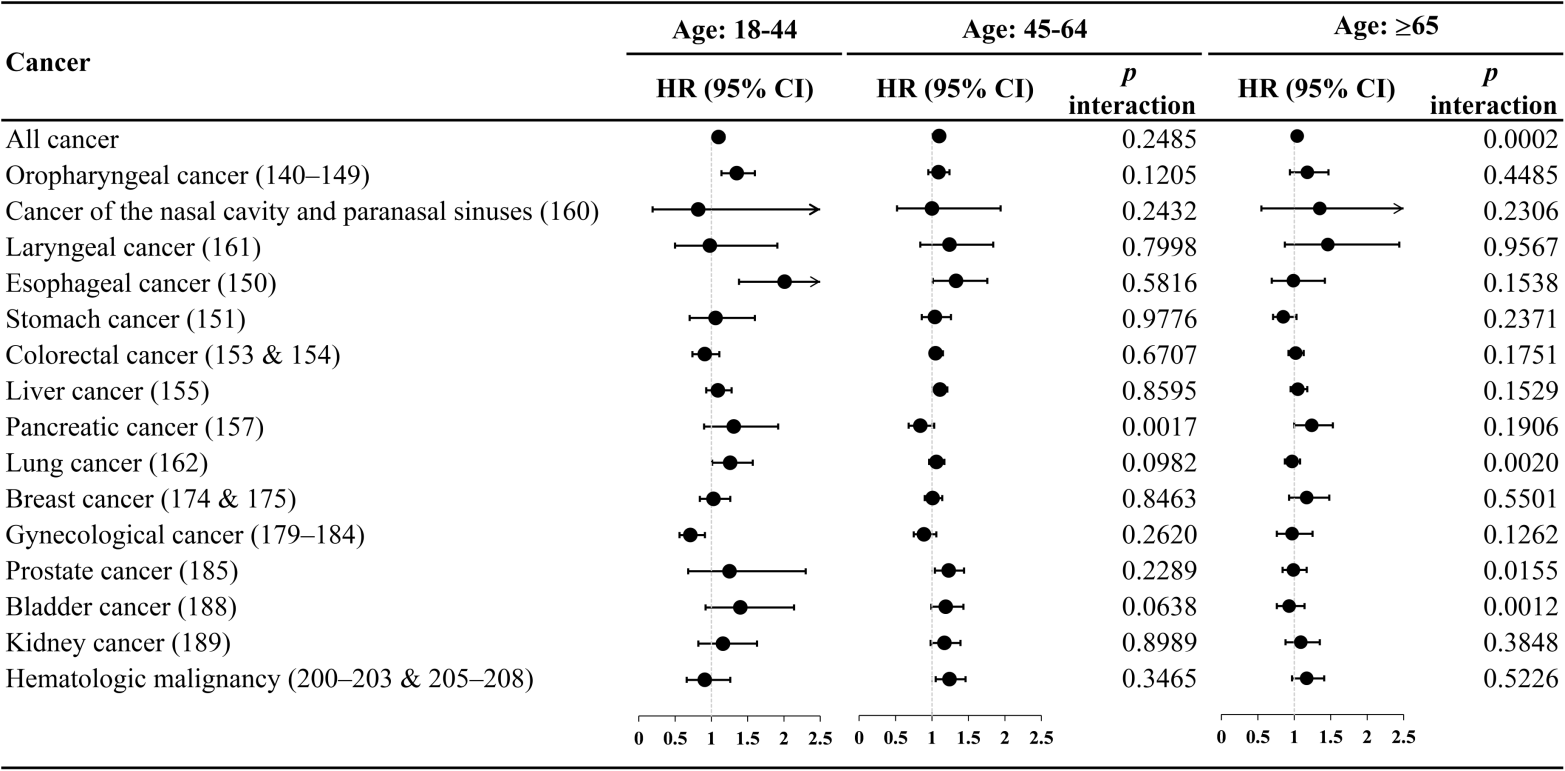
Hazard ratio of all cancers and site‐specific cancer for depression in patients with diabetes, stratified by age groups.

Considering subgroup analysis for sex and age groups (Figure 4 and Figure 5, detailed numbers in Table S5), we found that the association of depression with oropharyngeal cancer, larynx cancer, and liver cancer among female patients with diabetes was stronger than that among male patients. Additionally, we found that the cancer risk of depression was higher among young diabetes patients for all cancers and site‐specific lung, prostate, and bladder cancer than among those aged ≥65 years.

## 4 DISCUSSION

In the present study, we found that patients with coexisting depression and diabetes had a higher cancer incidence than those with diabetes only. Regarding site‐specific cancer, depression was associated with oropharyngeal cancer, esophageal cancer, liver cancer, prostate cancer, kidney cancer, and hematologic malignancy among patients with diabetes. A severity‐response relation was observed, i.e., patients with recurrent episodes of major depressive disorders had higher cancer incidence compared to those with dysthymia or depressive disorder not otherwise specified. However, there was no relationship between adherence to antidepressant treatment and cancer risk.

To our knowledge, this is the first study to examine the association between depression and cancer incidence in a diabetic cohort. One recent meta‐analysis study, not limited to patients with diabetes, demonstrated that depression is associated with a 1.13‐fold (95% CI 1.06–1.19) increased risk for overall cancer incidence.^11^ Our study showed a 1.08‐fold increased risk of overall cancer incidence, which was slightly lower than that of previous reports.^11^, ^33^ This difference might be because our study population included all unipolar depressive disorders, including major depressive disorder, dysthymic disorder, and depression disorder NOS. The association between depression and cancer risk may be attenuated by minor depression.

The increased risk of cancer incidence among patients with comorbid depression and diabetes may be explained by several factors. First, patients with depressive disorders, especially those with severe symptoms, might have poor glycemic control.^15^, ^34^ Hyperglycemia might further increase the risk of cancer incidence.^35^ Additionally, patients with depression might have less physical activity, and unhealthy dietary choices,^36^ which are related to increased risk of cancer incidence.^37^ Finally, depression is associated with changes in the inflammatory process and immune system,^38^ thereby further increasing the risk of diabetes complications^39^ and cancer development.^40^

Considering the analysis of site‐specific cancer, our findings were consistent with those of previous studies not limited to diabetes patients, which demonstrated that depression was associated with an increased risk of developing oropharynx cancer,^41^, ^42^ esophageal cancer,^42^ liver cancer,^42^, ^43^ prostate cancer,^11^, ^42^ kidney cancer,^11^, ^42^ and hematologic malignancy.^11^ Individuals with depressive disorders may exhibit unhealthy behaviors, including smoking and excessive alcohol consumption. These behaviors are associated with an elevated risk of oropharyngeal, esophageal, liver, and prostate cancer.^44^, ^45^ The linkage between depression and hematological malignancy remains less elucidated. Potential mechanisms such as alterations in cortisol levels and stress‐related hormones could play a role.^46^ Unexpectedly, we found that depression was associated with a reduced risk of gynecological cancer. A recent meta‐analysis showed no association between depression and gynecological cancer.^11^ Further investigation should replicate this finding and explore the effect of depression on potential risk factors, such as sexual behavior and hormone replacement therapy.

Of note, we found that adherence to antidepressant treatment did not alter the association between depression and cancer risk among patients with diabetes, except for larynx cancer. One meta‐analysis study demonstrated that the pooled odds ratio of antidepressant use for the risk of breast/ovarian cancer in epidemiologic studies was 1.11 (95% CI, 1.03–1.20).^47^ However, several recent epidemiological studies have reported null findings.^48^, ^49^ Some studies have demonstrated that antidepressant use reduces the risk of colorectal cancer;^20^, ^50^ this has been replicated by other studies.^51^, ^52^

Considering effect modifications by sex and age, the overall cancer incidence was not modified by sex; however, young adults (20–44 years) had a higher cancer risk than older adults (65 years). Previous research, including our own study and a recent meta‐analysis, has shown that the association between depression and macrovascular complications is stronger among young adults than older adults.^9^, ^53^ This difference in magnitude of association could be attributed to the fact that older adults tend to accumulate multiple risk factors, which may attenuate the relative risk of depression. Conversely, among young adults, depression could be among the few but significant risk factors for cancer incidence.

### 4.1 Study limitations

This study had several limitations. Firstly, there is a possibility that some patients with depression were undiagnosed or untreated, leading to their classification in the comparison group. Such misclassification could underestimate the association between depression and cancer incidence. Secondly, the accuracy of cancer diagnoses based on ambulatory and inpatient records in the NHI claims database has not been completely validated. However, we believe that any potential misclassification is likely nondifferential between diabetic patients with and without depression, thereby resulting in bias towards null findings. In addition, the cancer stage information in the NHI claims database is absent; we could not investigate the potential influence of comorbid depression on the stage of cancer diagnosis. Thirdly, the impact of diabetes courses, as well as the types of antidepressant and antidiabetic drugs, on their potential association with cancer risk remains unexamined, necessitating further investigation. Fourthly, confounding covariates were identified in the year prior to the onset of diabetes. Therefore, the temporal relationship between covariates and depression was not clear. Nevertheless, the impact on the study results might be relatively minor, as the study aimed to compare cancer risk between incident diabetes patients with and without depression. Fifthly, we employed prescription records to measure medication adherence; nevertheless, we could not definitively determine whether patients ultimately consumed the prescribed medication. Finally, important lifestyle factors such as diet, body weight, smoking, and exercise were unavailable in the NHI claims database. We used proxy measures such as dyslipidemia, hypertension, chronic pulmonary disease, and alcohol or substance use disorders. However, residual confounding factors may have influenced our findings.

### 4.2 Clinical implications

Our study provides evidence that this comorbidity of depression may increase the risk of cancer incidence in patients with diabetes. Cancer screening and monitoring should be given close attention to this patient population. Moreover, our findings indicate an association between depression and site‐specific cancers, such as oropharyngeal, esophageal, liver, prostate, kidney, and hematologic malignancies, in patients with diabetes. This highlights the need for heightened vigilance for these types of cancers in this patient group. While adherence to antidepressant treatment was not found to be related to cancer risk, the effective management of depression remains critical for enhancing the overall health outcomes of patients with diabetes.

### 4.3 Conclusions

We found that patients with coexisting diabetes and depression were associated with an increased risk of cancer incidence than those with diabetes only. Antidepressant treatment was not associated with cancer risk. Future investigations should replicate our findings, explore the effect of non‐pharmacological treatment for depression on cancer risk, and identify the underlying mechanisms.

## AUTHOR CONTRIBUTIONS

**Wang Shi‐Heng:** Conceptualization (equal); formal analysis (equal); writing – original draft (equal); writing – review and editing (equal). **Le‐Yin Hsu:** Data curation (equal); methodology (equal). **Mei‑Chen Lin:** Data curation (lead); formal analysis (lead). **Chi‐Shin Wu:** Conceptualization (equal); writing – original draft (equal); writing – review and editing (equal).

## FUNDING INFORMATION

This work was partly supported by the Ministry of Science and Technology (MOST 104‐2321‐B‐039‐007‐MY3, SHW) and the National Health Research Institutes, Taiwan (CG‐110‐GP‐01 & CG‐111‐GP‐01, CSW). The funding agency had no role in study design, data collection and analysis, decision to publish, or manuscript preparation.

## CONFLICT OF INTEREST STATEMENT

The authors declare that there is no conflict of interest.

## Supporting information


Table S1.
Table S2.
Table S3.
Table S4.
Table S5.

## Data Availability

The Taiwan's NHI database cannot be made available due to the Personal Information Protection Act executed by Taiwan's government. Requests for data can be sent as a formal proposal to the Ministry of Health and Welfare (https://www.nhi.gov.tw/English/).
