# Supplementary material for: Associations between depression and cancer risk among patients with diabetes mellitus: A population‐based cohort study
Source: Cancer Med. 2023 Sep 14;12(19):19968–77. doi: 10.1002/cam4.6539 (PMC10587979; doi:10.1002/cam4.6539)
Supplement: Supplementary file 1 — Table S1. Table S2. Table S3. Table S4. Table S5. [file CAM4-12-19968-s001.docx]

Supplementary Table 1. Baseline characteristics in the year before entry and anti-diabetic medication MPR in the year after entry

|  | DM & MDD group  (n=85,489) | comparison group  (n=427,445) | *p*-value |
| --- | --- | --- | --- |
| Sex, Female | 52,761 (61.7) | 263,805 (61.7) | - |
| Age, mean ± SD | 51.2 ±13.8 | 51.2 ±13.8 | - |
| Age |  |  | - |
| 20-44 | 27,868 (32.6) | 139,340 (32.6) |  |
| 45-64 | 41,141 (48.1) | 205,705 (48.1) |  |
| >=65 | 16,480 (19.3) | 82,400 (19.3) |  |
| **Comorbidity** |  |  |  |
| 1 Hypertension | 46,510 (54.4) | 203,442 (47.6) | <.0001 |
| 2 Dyslipidemia | 27,516 (32.2) | 110,222 (25.8) | <.0001 |
| 3 Chronic liver diseases | 395 (0.5) | 925 (0.2) | <.0001 |
| 4 Chronic pulmonary diseases | 13,748 (16.1) | 44,215 (10.3) | <.0001 |
| 7 SCZ | 3,715 (4.4) | 2,829 (0.7) | <.0001 |
| 8 BP | 3,674 (4.3) | 970 (0.2) | <.0001 |
| 5 Anxiety disorder | 28,788 (33.7) | 34,380 (8.0) | <.0001 |
| 6 Alcohol/substance use | 3,452 (4.0) | 4,017 (0.9) | <.0001 |
| **Medication use** |  |  |  |
| ACEI/ARB | 5,739 (6.7) | 33,138 (7.8) | <.0001 |
| Beta blocker | 6,176 (7.2) | 26,575 (6.2) | <.0001 |
| Calcium channel blocker | 5,613 (6.6) | 29,658 (6.9) | <.0001 |
| Diuretics | 5,431 (6.4) | 21,814 (5.1) | <.0001 |
| Lipid-lowering agent | 9,761 (11.4) | 52,226 (12.2) | <.0001 |
| Antipsychotics | 6,952 (8.1) | 22,562 (5.3) | <.0001 |
| NSAID | 9,916 (11.6) | 61,397 (14.4) | <.0001 |
| **Healthy system utilization** |  |  |  |
| Out-patient visit |  |  | <.0001 |
| <10 | 10,900 (12.8) | 149,697 (35.0) |  |
| 10-19 | 21,322 (24.9) | 133,988 (31.4) |  |
| >=20 | 53,267 (62.3) | 143,760 (33.6) |  |
| Admission to hospital | 22,005 (25.7) | 79,403 (18.6) | <.0001 |
| **Anti-diabetic medication MPR** |  |  | <.0001 |
| <=0.2 | 28,480 (33.3) | 131,258 (30.7) |  |
| 0.2–0.8 | 42,583 (49.8) | 227,377 (53.2) |  |
| >0.8 | 14,426 (16.9) | 68,810 (16.1) |  |

Supplementary Table 2. Events and person-years for all cancer and site-specific cancer in two cohorts.

|  | Patients with depression and diabetes (n=85,489) | | Patients with diabetes only  (n=427,445) | |
| --- | --- | --- | --- | --- |
|  | Event | person-years | Event | person-years |
| All cancer | 8,157 | 524,148 | 35,867 | 2,688,523 |
| Oropharyngeal cancer (140–149) | 644 | 545,897 | 2,558 | 2,776,005 |
| Cancer of the nasal cavity and paranasal sinuses (160) | 171 | 547,379 | 571 | 2,782,122 |
| Laryngeal cancer (161) | 324 | 546,969 | 1,692 | 2,779,610 |
| Esophageal cancer (150) | 1,378 | 543,184 | 6,506 | 2,764,734 |
| Stomach cancer (151) | 1,575 | 544,092 | 6,565 | 2,769,638 |
| Colorectal cancer (153 & 154) | 291 | 547,316 | 1,291 | 2,781,119 |
| Liver cancer (155) | 20 | 547,701 | 90 | 2,782,895 |
| Pancreatic cancer (157) | 83 | 547,468 | 294 | 2,782,340 |
| Lung cancer (162) | 1,226 | 544,945 | 5,469 | 2,771,912 |
| Breast cancer (174 & 175) | 617 | 545,589 | 2,903 | 2,773,789 |
| Gynecological cancer (179–184) | 356 | 546,566 | 2,139 | 2,776,325 |
| Prostate cancer (185) | 440 | 546,342 | 1,853 | 2,777,320 |
| Bladder cancer (188) | 341 | 546,755 | 1,444 | 2,779,244 |
| Kidney cancer (189) | 366 | 546,724 | 1,490 | 2,779,327 |
| Hematologic malignancy (200–203 & 205–208) | 397 | 546,816 | 1,631 | 2,779,424 |

Supplementary Table 3. Depression and cancer risk in patients with diabetes, stratified by depression subtype

|  | Major depressive disorder, with recurrent episodes (n=20,140) | Major depressive disorder, with single episode (n=13,442) | Dysthymia (n=43,441) | Depressive disorder, NOS (n=8,466) | types of MDD *MDD interaction  (single vs recurrent)  (Dysthymia vs recurrent)  (NOS vs recurrent) |
| --- | --- | --- | --- | --- | --- |
| All cancer | 1.15  (1.08-1.22) | 1.15  (1.07-1.23) | 1.05  (1.01-1.09) | 1.02  (0.94-1.11) | p=0.9124  p=0.0092  p=0.0132 |
| Oropharyngeal cancer (140–149) | 1.21  (0.98-1.50) | 1.03  (0.79-1.34) | 1.22  (1.07-1.38) | 1.12  (0.83-1.52) | p=0.5924  p=0.6998  p=0.5311 |
| Cancer of the nasal cavity and paranasal sinuses (160) | 0.91  (0.30-2.76) | 1.07  (0.19-6.15) | 1.26  (0.67-2.36) | - | p=0.8106  p=0.7406  - |
| Laryngeal cancer (161) | 0.72  (0.37-1.40) | 1.81  (0.86-3.80) | 1.31  (0.91-1.88) | 1.20  (0.46-3.11) | p=0.0780  p=0.2192  p=0.7987 |
| Esophageal cancer (150) | 1.33  (0.87-2.01) | 1.59  (1.02-2.48) | 1.26  (0.96-1.65) | 1.41  (0.76-2.61) | p=0.1650  p=0.6154  p=0.3847 |
| Stomach cancer (151) | 0.83  (0.61-1.14) | 1.15  (0.83-1.58) | 0.94  (0.80-1.12) | 0.95  (0.64-1.41) | p=0.2772  p=0.6884  p=0.6870 |
| Colorectal cancer (153 & 154) | 1.17  (1.03-1.34) | 1.18  (1.01-1.39) | 0.97  (0.89-1.05) | 0.89  (0.72-1.10) | p=0.5815  p=0.0039  p=0.0140 |
| Liver cancer (155) | 1.11  (0.97-1.27) | 1.12  (0.96-1.31) | 1.11  (1.02-1.20) | 1.00  (0.82-1.21) | p=0.7699  p=0.8922  p=0.3909 |
| Pancreatic cancer (157) | 1.16  (0.85-1.57) | 1.13  (0.82-1.58) | 0.90  (0.74-1.09) | 1.50  (0.99-2.28) | p=0.9694  p=0.0427  p=0.6951 |
| Lung cancer (162) | 1.07  (0.91-1.25) | 1.07  (0.91-1.27) | 1.00  (0.91-1.09) | 1.18  (0.97-1.44) | p=0.7028  p=0.7236  p=0.5341 |
| Breast cancer (174 & 175) | 1.20  (1.00-1.45) | 1.07  (0.85-1.35) | 0.93  (0.81-1.06) | 1.16  (0.88-1.55) | p=0.2681  p=0.0028  p=0.4500 |
| Gynecological cancer (179–184) | 0.71  (0.54-0.94) | 0.76  (0.55-1.05) | 0.91  (0.77-1.07) | 1.19  (0.84-1.70) | p=0.2133  p=0.0232  p=0.0060 |
| Prostate cancer (185) | 1.32  (1.01-1.72) | 1.09  (0.82-1.45) | 1.09  (0.94-1.27) | 1.10  (0.80-1.53) | p=0.9561  p=0.5765  p=0.8664 |
| Bladder cancer (188) | 0.98  (0.72-1.34) | 1.59  (1.17-2.16) | 1.05  (0.88-1.25) | 1.02  (0.70-1.48) | p=0.0172  p=0.4554  p=0.6227 |
| Kidney cancer (189) | 1.19  (0.89-1.58) | 1.33  (0.97-1.82) | 1.13  (0.95-1.33) | 0.86  (0.57-1.29) | p=0.5100  p=0.7598  p=0.1524 |
| Hematologic malignancy (200–203 & 205–208) | 1.37  (1.06-1.78) | 1.18  (0.88-1.59) | 1.06  (0.90-1.25) | 1.21  (0.85-1.72) | p=0.3959  p=0.0613  p=0.4616 |

Supplementary Table 4. Depression and cancer risk in patients with diabetes, stratified by antidepressant MPR

|  | MPR<0.2  (n=62,085) | MRP 0.2-0.8 (n=15,890) | MRP >=0.8 (n=7,514) | antidepressant MPR *MDD interaction  (0.2-0.8 vs <0.2)  (≥0.8 vs <0.2) |
| --- | --- | --- | --- | --- |
| All cancer | 1.08  (1.04-1.11) | 1.11  (1.04-1.18) | 1.06  (0.96-1.17) | p=0.7121  p=0.6398 |
| Oropharyngeal cancer (140–149) | 1.19  (1.07-1.33) | 1.11  (0.88-1.39) | 1.23  (0.86-1.77) | p=0.3888  p=0.8970 |
| Cancer of the nasal cavity and paranasal sinuses (160) | 0.97  (0.56-1.68) | 1.41  (0.35-5.73) | 0.44  (0.15-1.31) | p=0.8803  p=0.7825 |
| Laryngeal cancer (161) | 1.41  (1.04-1.91) | 1.06  (0.50-2.26) | 0.42  (0.08-2.08) | p=0.3811  p=0.0298 |
| Esophageal cancer (150) | 1.23  (0.97-1.56) | 1.63  (1.05-2.54) | 1.63  (0.93-2.83) | p=0.6891  p=0.4216 |
| Stomach cancer (151) | 0.90  (0.78-1.04) | 1.22  (0.89-1.66) | 0.92  (0.56-1.51) | p=0.1864  p=0.8108 |
| Colorectal cancer (153 & 154) | 1.02  (0.95-1.10) | 1.10  (0.94-1.28) | 0.93  (0.74-1.18) | p=0.4627  p=0.9083 |
| Liver cancer (155) | 1.10  (1.03-1.18) | 1.11  (0.96-1.29) | 1.07  (0.85-1.34) | p=0.6094  p=0.7632 |
| Pancreatic cancer (157) | 1.05  (0.90-1.23) | 1.05  (0.75-1.48) | 1.00  (0.59-1.69) | p=0.5103  p=0.1942 |
| Lung cancer (162) | 1.06  (0.98-1.15) | 0.96  (0.82-1.14) | 1.00  (0.78-1.28) | p=0.3187  p=0.6759 |
| Breast cancer (174 & 175) | 1.00  (0.90-1.11) | 1.12  (0.89-1.42) | 1.21  (0.88-1.66) | p=0.1593  p=0.1968 |
| Gynecological cancer (179–184) | 0.91  (0.80-1.05) | 0.71  (0.52-0.97) | 0.77  (0.49-1.21) | p=0.0640  p=0.1374 |
| Prostate cancer (185) | 1.10  (0.96-1.25) | 1.20  (0.92-1.57) | 1.22  (0.78-1.93) | p=0.5540  p=0.5607 |
| Bladder cancer (188) | 1.10  (0.95-1.27) | 1.11  (0.78-1.56) | 1.20  (0.72-1.98) | p=0.9824  p=0.7349 |
| Kidney cancer (189) | 1.14  (0.99-1.31) | 1.23  (0.90-1.68) | 1.00  (0.63-1.60) | p=0.8890  p=0.8992 |
| Hematologic malignancy (200–203 & 205–208) | 1.13  (0.98-1.29) | 1.16  (0.89-1.52) | 1.38  (0.89-2.14) | p=0.2418  p=0.6639 |

Supplementary Table 5. Depression and cancer risk in patients with diabetes, stratified by sex and age groups

|  | male | female | Sex*MDD interaction | Age: 18-44 | Age: 45-64 | Age: >=65 | Age*MDD interaction  (45-64 vs 18-44)  ( >=65 vs 18-44) |
| --- | --- | --- | --- | --- | --- | --- | --- |
| All cancer | 1.08  (1.04-1.12) | 1.09  (1.05-1.12) | p=0.1147 | 1.10  (1.03-1.18) | 1.10  (1.06-1.14) | 1.04  (1.00-1.09) | p=0.2485  p=0.0002 |
| Oropharyngeal cancer (140–149) | 1.14  (1.01-1.28) | 1.26  (1.08-1.48) | p=0.0179 | 1.35  (1.14-1.60) | 1.09  (0.95-1.24) | 1.18  (0.94-1.47) | p=0.1205  p=0.4485 |
| Cancer of the nasal cavity and paranasal sinuses (160) | 0.83  (0.38-1.83) | 1.23  (0.64-2.38) | p=0.3878 | 0.82  (0.19-3.49) | 1.00  (0.52-1.94) | 1.35  (0.55-3.33) | p=0.2432  p=0.2306 |
| Laryngeal cancer (161) | 1.11  (0.80-1.53) | 1.98  (1.16-3.40) | p=0.0288 | 0.98  (0.50-1.91) | 1.24  (0.84-1.84) | 1.46  (0.87-2.44) | p=0.7998  p=0.9567 |
| Esophageal cancer (150) | 1.33  (1.06-1.67) | 1.31  (0.92-1.85) | p=0.4497 | 2.01  (1.38-2.95) | 1.33  (1.01-1.76) | 0.99  (0.69-1.42) | p=0.5816  p=0.1538 |
| Stomach cancer (151) | 0.90  (0.75-1.08) | 1.01  (0.84-1.20) | p=0.4302 | 1.06  (0.70-1.60) | 1.04  (0.86-1.26) | 0.85  (0.71-1.03) | p=0.9776  p=0.2371 |
| Colorectal cancer (153 & 154) | 1.00  (0.90-1.10) | 1.06  (0.97-1.15) | p=0.4635 | 0.91  (0.74-1.11) | 1.05  (0.97-1.15) | 1.02  (0.92-1.13) | p=0.6707  p=0.1751 |
| Liver cancer (155) | 1.04  (0.96-1.14) | 1.16  (1.07-1.26) | p=0.0006 | 1.09  (0.93-1.28) | 1.11  (1.03-1.21) | 1.05  (0.95-1.18) | p=0.8595  p=0.1529 |
| Pancreatic cancer (157) | 0.99  (0.79-1.24) | 1.08  (0.91-1.29) | p=0.3282 | 1.31  (0.90-1.92) | 0.84  (0.68-1.03) | 1.24  (1.00-1.53) | p=0.0017  p=0.1906 |
| Lung cancer (162) | 1.03  (0.93-1.13) | 1.07  (0.97-1.17) | p=0.1403 | 1.26  (1.01-1.57) | 1.06  (0.96-1.17) | 0.97  (0.87-1.08) | p=0.0982  p=0.0020 |
| Breast cancer (174 & 175) | 2.03  (0.77-5.37) | 1.04  (0.94-1.14) | p=0.1778 | 1.03  (0.84-1.26) | 1.01  (0.90-1.14) | 1.17  (0.93-1.48) | p=0.8463  p=0.5501 |
| Gynecological cancer (179–184) | - | 0.86  (0.76-0.97) | - | 0.71  (0.56-0.91) | 0.89  (0.75-1.06) | 0.97  (0.76-1.25) | p=0.2620  p=0.1262 |
| Prostate cancer (185) | 1.12  (1.00-1.26) | - | - | 1.25  (0.68-2.30) | 1.23  (1.04-1.44) | 0.99  (0.84-1.17) | p=0.2289  p=0.0155 |
| Bladder cancer (188) | 1.02  (0.85-1.22) | 1.20  (1.00-1.43) | p=0.0598 | 1.40  (0.92-2.14) | 1.19  (0.99-1.43) | 0.93  (0.76-1.14) | p=0.0638  p=0.0012 |
| Kidney cancer (189) | 1.16  (0.95-1.43) | 1.13  (0.96-1.32) | p=0.8175 | 1.16  (0.82-1.63) | 1.17  (0.98-1.39) | 1.09  (0.88-1.35) | p=0.8989  p=0.3848 |
| Hematologic malignancy (200–203 & 205–208) | 1.23  (1.03-1.47) | 1.11  (0.95-1.30) | p=0.4343 | 0.91  (0.66-1.26) | 1.24  (1.05-1.46) | 1.17  (0.97-1.41) | p=0.3465  p=0.5226 |
